# An ensemble learning model for forecasting water-pipe leakage

**DOI:** 10.1038/s41598-024-60840-x

**Published:** 2024-05-09

**Authors:** Ahmed Ali Mohamed Warad, Khaled Wassif, Nagy Ramadan Darwish

**Affiliations:** 1 Department of Information Systems and Technology, Faculty of Graduate Studies for Statistical Research, Cairo University, Cairo, Egypt; 2 Department of Computer Science, Faculty of Computers and Artificial Intelligence, Cairo University, Cairo, Egypt

**Keywords:** Information theory and computation, Engineering, Mathematics and computing, Computational science, Computer science, Information technology, Scientific data

## Abstract

Based on the benefits of different ensemble methods, such as bagging and boosting, which have been studied and adopted extensively in research and practice, where bagging and boosting focus more on reducing variance and bias, this paper presented an optimization ensemble learning-based model for a large pipe failure dataset of water pipe leakage forecasting, something that was not previously considered by others. It is known that tuning the hyperparameters of each base learned inside the ensemble weight optimization process can produce better-performing ensembles, so it effectively improves the accuracy of water pipe leakage forecasting based on the pipeline failure rate. To evaluate the proposed model, the results are compared with the results of the bagging ensemble and boosting ensemble models using the root-mean-square error (RMSE), the mean square error (MSE), the mean absolute error (MAE), and the coefficient of determination (R2) of the bagging ensemble technique, the boosting ensemble technique and optimizable ensemble technique are higher than other models. The experimental result shows that the optimizable ensemble model has better prediction accuracy. The optimizable ensemble model has achieved the best prediction of water pipe failure rate at the 14th iteration, with the least RMSE = 0.00231 and MAE = 0.00071513 when building the model that predicts water pipe leakage forecasting via pipeline failure rate.

## Introduction

In recent years, artificial intelligence (AI) and machine learning (ML) models have been suggested to be revolutionary innovations^1^. ML is a branch of artificial intelligence that collects methods and algorithms for building experience-based learning systems. On the other side, Water supply system leakage is a quiet problem that costs the globe billions of dollars each year. Because a large portion of the water supply pipelines are underground, leaks might go unnoticed and unreported for a long period of time^2^. Regarding water supply networks, there is a global trend among service management organizations to use machine learning to forecast pipe problems and breakages. So, ML has been used to forecast Water pipe leakage of the water distribution network (WDN), with research on data validation and enhancement as well as investigations on the relationships between intervening factors that might explain the intricate process of pipe failure^2^. In our previous work presented a systematic literature review (SLR) that employs ML models for water leakage problem^3^.Various studies have revealed the importance of water pipe leakage forecasting and presented machine learning algorithms for forecasting water pipe leakage and its failure rate. These studies include some of the most popular statistical models, such as linear regression (LR), poison regression (PR), and evolutionary polynomial regression (EPR). As machine-learning techniques, they use gradient boost trees (GB)^4–7^, Bayesian belief networks^8–10^, Support Vector Machines (SVMs)^11–13^ and Artificial Neural Networks (ANNs)^11,14–19^, These studies have consistently found that ML models can provide valuable insights into the condition of these pipelines and help prioritize maintenance, and repair efforts based on forecasting the failure of water pipes; however, ensemble approaches as a machine learning technique for water pipe leakage predictions have yet to be thoroughly investigated.

Several ensemble models and approaches have been devised and widely utilized for classification and regression issues over the last two decades. In data analytics, ensemble models^20^ are well-motivated, but not all ensembles are created equal. Specifically, different types of ensembles include bagging, and boosting. Each strategy has advantages and disadvantages. Bagging tends to decrease variance, not bias, to solve the over-fitting problem boosting aims to decrease bias, not variance by sequentially combining weak learners but is sensitive to noisy data and outliers and is prone to overfitting, as shown in Table 1.

**Table 1:** Comparation between boosting and bagging techinque.

	Boosting	Bagging
The aim of the model	To decrease bias, not variance	To decrease variance not bias, to solve the over-fitting problem
Type of combing predictions	Different types	The same type of prediction
The weight of layer models	According to their performance	Each model has the same weightage
Training data subsets	Every new data subset contains the elements were misclassified by previous models	Randomly drawn with replacement from the entire training dataset
The independent between the models	New Models are influenced by the accuracy of previous Models (sequential)	Each model is independent of each other (parallel)

Ensemble learning methods^21^ have been widely used in various applications and areas, from healthcare^22^, finance^23,24^, image recognition^25^, natural language processing^26–28^, enabling informed decision-making and predictive analytics^29,30^. To fit ensemble learning models into different problems, their hyperparameters must be tuned. Selecting the best hyperparameter configuration for ensemble learning models has a direct impact on the model’s performance^31^. On the other side, pipe failure is an essential instrument for water distribution network strategic restoration planning. Existing network data (physical data) and historical failure records (number of breaks) are used to make rehabilitation projections.^32^. Subsequently, the pipe failure rate is an important measure to water pipe leakage forecasting.

The aim of this paper is to suggest a new model, that focuses on optimization ensemble weights and hyperparameter ensemble methods in regression problems, which is one challenging part of constructing an optimal ensemble, with the purpose of forecasting water pipe leakage using the failure rate of water pipes by integrating the best hyperparameter tuning of ensemble learning regression methods. The proposed model involves collecting a dataset for water pipe leakage. This dataset includes several features linked to pipeline failures, such as pipeline material, age, etc. Pre-processing, feature selection, and descriptive statistical analysis are performed on the dataset that was collected from Alexandria Water Company Egypt (AWCO). The proposed model used the Bayesian optimization method for optimizing the weights and hyperparameters of ensemble learning for the water pipe leakage problem. Next, compare the optimization ensemble method, boosted tree ensemble learning, and bagged tree ensemble learning. Each model's performance varies based on the dataset and the model's base learner, with Bayesian parameter optimization producing the most accurate predictions.

This paper is organized as follows: Section "Modelling techniques" discusses modelling techniques. In section "Proposed methodology for model development", the proposed methodology and model development are discussed, along with the procedural details required for water pipe leakage forecasting. The proposed model's performance is compared to bagging and boosting models, as explained in Section "Results and discussion". Finally, the paper's summary and recommendation for further research are provided in Section "Conclusion".

## Modelling techniques

Ensemble Learning^33,34^ is, one of the hot topics, the integration of numerous learners (classification and regression models) to build a powerful learner (ensemble model). Unlike traditional learning methods, which attempt to build a single model from training data, ensemble learning methods attempt to build numerous models to tackle the same issue. Due to the availability of precise and diversified multiple models for integrating into a single solution, ensemble learning typically gives solutions with higher accuracy and/or resilience in most situations. Ensemble learning is often done in three phases: (1) development of base models, (2) selection of base models, and (3) aggregation of the selected base models utilizing certain combination methods. In the first step, a pool of basic models is formed, which might be homogeneous (same model types) or heterogeneous (various model types) (mixture of different model types). A base learning algorithm, such as decision trees, neural networks, or other approaches, is typically used to build base learners from training data. A selection of basic models is chosen in the second step. Finally, using a combination approach, the selected models are aggregated to produce a model. An ensemble's generalization capacity is frequently substantially stronger than that of basic learners. To obtain the final model with greater generalization, it is critical that the basic models be as precise and varied as feasible.

### Bagging technique

**Figure 1:**
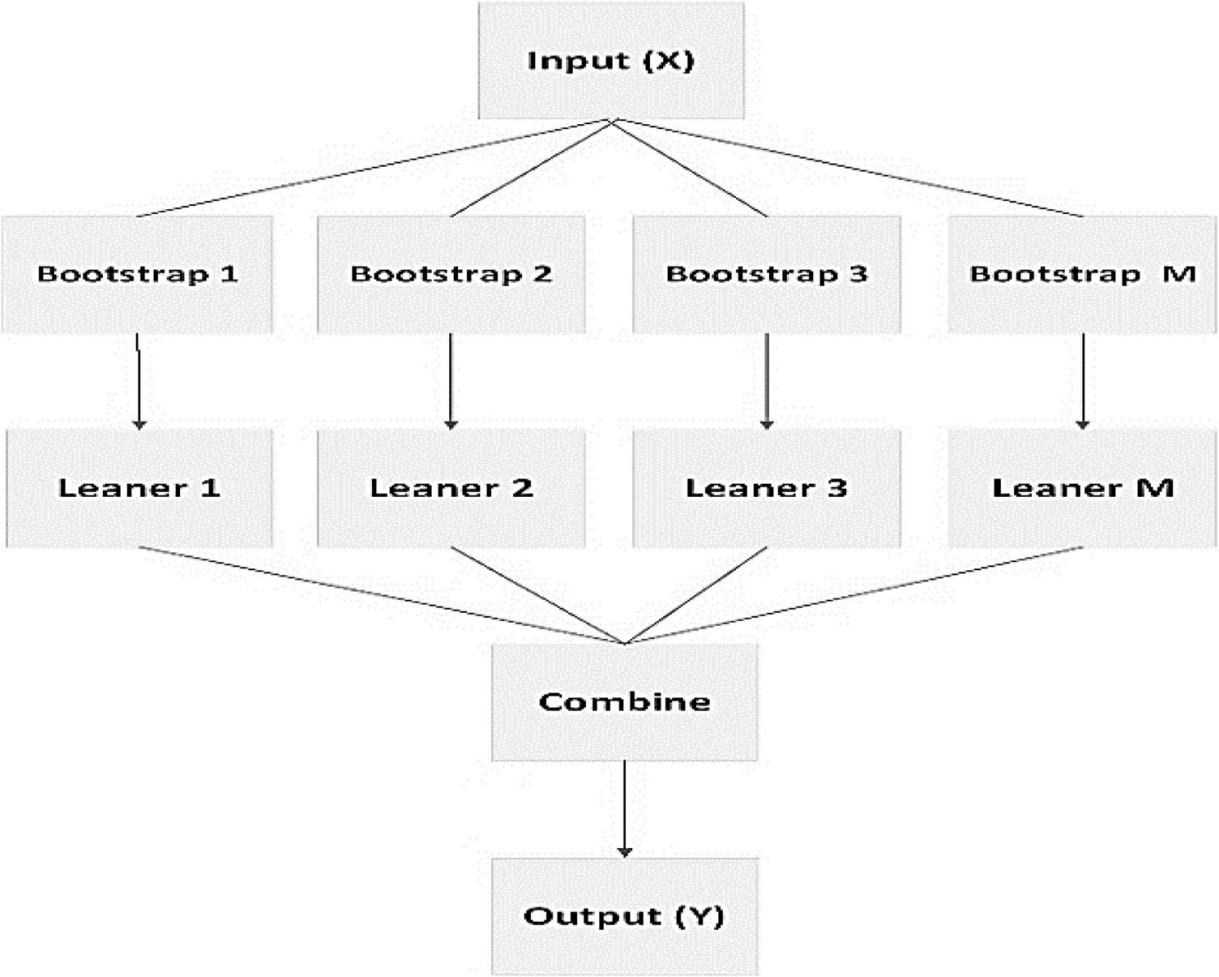
Bagging ensemble technique.

Bagging^33,35^ is an ensemble learning approach that is also known as Bootstrap aggregation. The same approach is used to train many models in parallel, each using a fraction of the training data created by bootstrap sampling. Bootstrap sampling is a sampling method in which a sample is formed by randomly picking items from a data collection and replacing them with replacement items. That is, after each selection, the item is returned to the data set. As a result, the same item may be picked more than once for the same sample. The metamodel is created by collecting the outcomes of many models by either voting (classification job) or averaging (regression task), as seen in Fig. 1.

Bagging is dependent on the varied training sizes of training data, which are referred to as bags, obtained from the training dataset. Each ensemble member is built using the tagging procedure. The prediction model is then constructed for each subset of bags, combining the values of several outputs by voting or averaging across the class label. The Bagging method first chooses a random sample with replacement from the original training dataset, and then generates numerous learner algorithm outputs (bags).

### Boosting technique

**Figure 2:**
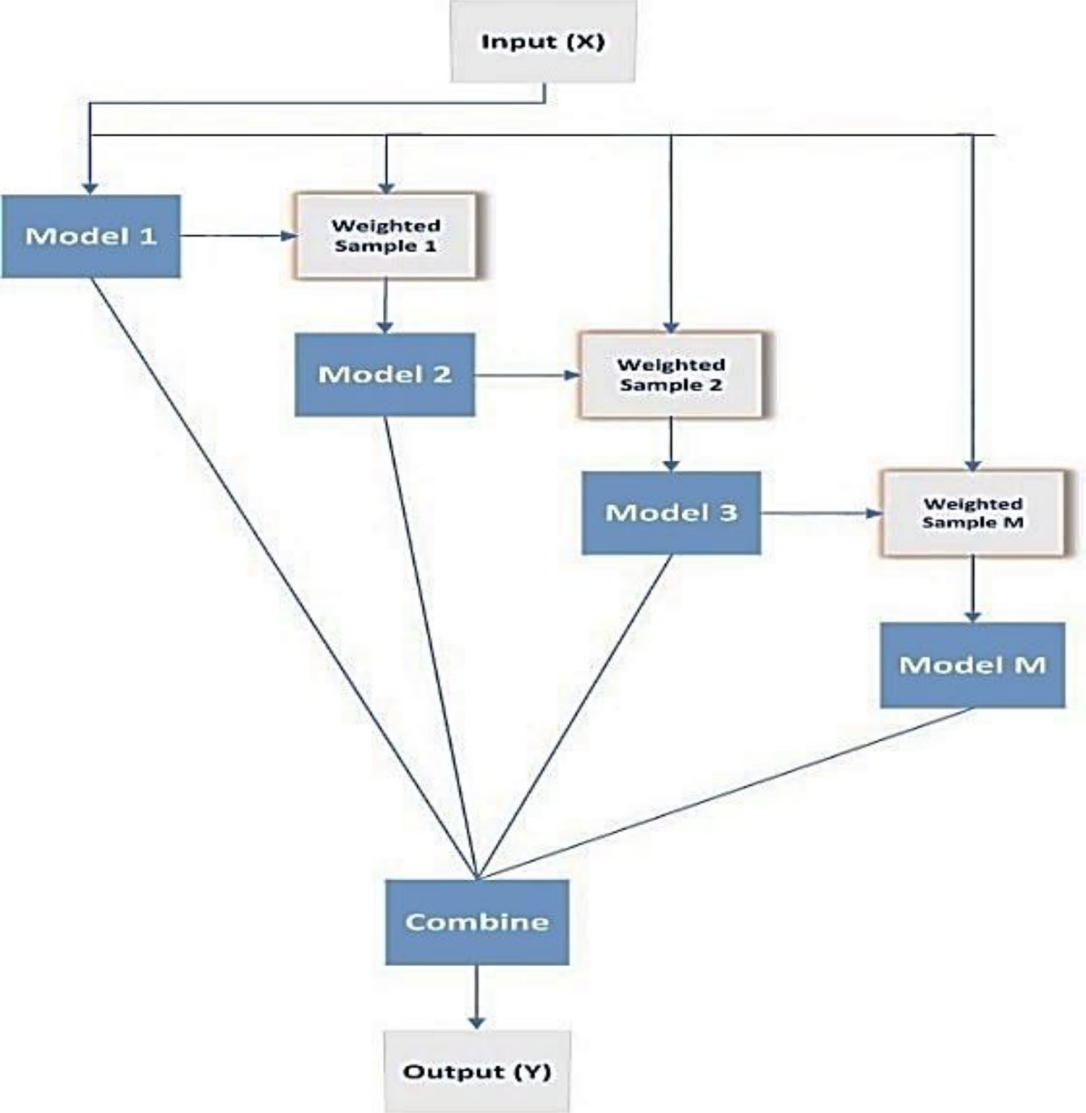
Boosting ensemble technique.

Boosting^34,36^ is a sequential ensemble method for converting low-accuracy models (weak learners) into strong ensemble models. After training a basic model with poor accuracy, the next generation of the model focuses on the instances in the training data set that were wrongly identified. Each succeeding model version is trained using the whole training data set to create an aggregated predictor, which reduces the likelihood of overfitting the data. Finally, using the weighted majority vote (classification task) or weighted sum, the predictions from each model are integrated into a single final forecast (regression task). Boosting, as seen in Fig. 2.

### Hyperparameter optimization model

Hyperparameter optimization^20,37^ is one of the major challenges in the ML industry. This stage includes identifying an effective hyperparameter configuration that enhances the model’s performance for a particular dataset. Usually, these hyperparameters are identified before beginning the learning process that are tuned based on the performance of the selected hyperparameter and a validation set performance as an objective.

**Figure 3:**
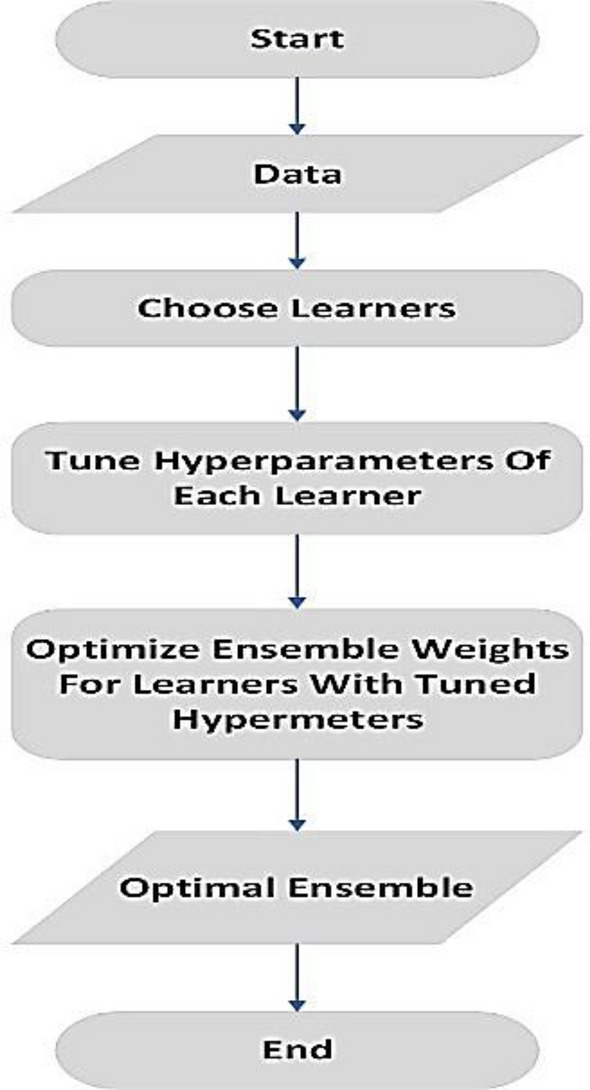
Ensemble model with internally tuned hyperparameters.

There are different hyperparameter optimization algorithms, such as (1) grid search is considered expensive from computationally side because require searching for all possible defined hyperparameter configurations to identify and select the optimal model, and (2) random search that try to overcome the limitations of the grid search by optimizing the model in a randomly selected hyperparameter configuration, however, its stochastic nature may result in a bad hyperparameter configuration, but (3) Bayesian optimization provide a surrogate solution by developing a probabilistic model and using an acquisition function that helps to identify the most probability hyperparameters incorporating the previous evaluations from the search space, as seen in Fig. 3.

In each iteration, Bayesian optimization seeks to gather observations with the maximum amount of information by striking a balance between exploitation and exploration (i.e., investigating unknown hyperparameters) (gathering observations from hyperparameters close to the optimum).

## Proposed methodology for model development

The proposed methodology is to develop a predictive model for water pipe leakage via pipeline failure rate using ensemble learning methods. Our method consists of the subsequent stages: (1) Dataset generation stage based on Alexandria Water Company (ACWA) as water supply systems in Alexandria, Egypt, and (2) the proposed model has developed three models including Bagging, Boosting, and optimizable ensemble methods in order to select the one with satisfactory performance for water leakage forecasting, and evaluated by RMSE, MSE, MAE, and R2. In addition, validated based on the real data collected. These stages will be explained more in the following sections.

### Dataset generation (case study: City of Alexandria)

Data is definitely the most vital element of machine learning. If there is no data, there is no common purpose. So, the aim of the collected data is to define the problem. Also, the way data is stored and organized is important based on the type of variable.

**Figure 4:**
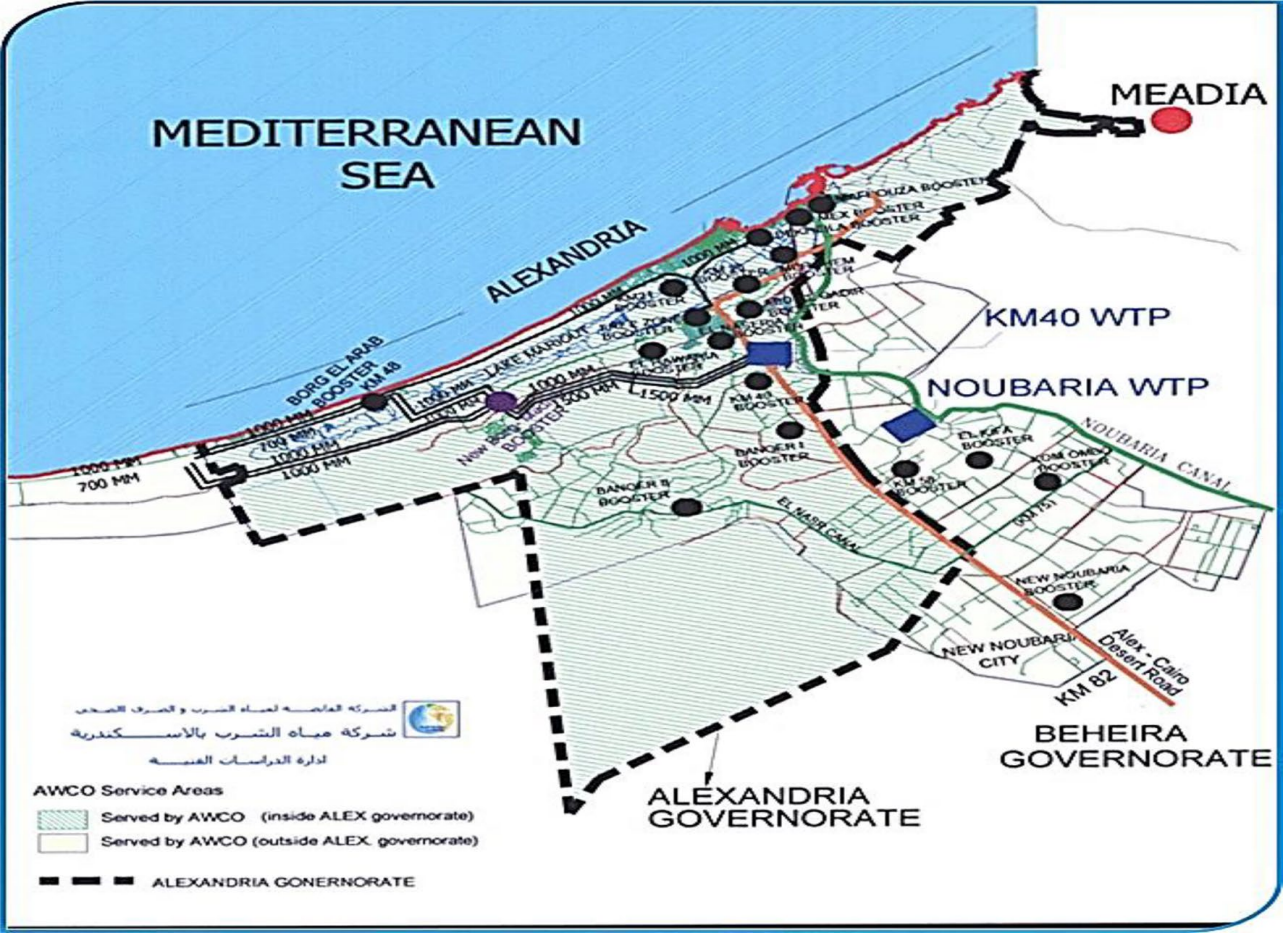
The water network of the city of Alexandria.

Using data from our research collected from water supply systems in Alexandria, Egypt, the cadastral base investigated has 1951913 water service connections and a length of distribution network of 9373 kilometers (Km), consisting of different types of materials such as "high-density polyethylene", "cast iron", and "polyvinyl chloride". Since the 1960s, the city of Alexandria has developed its water distribution network as part of its infrastructure, that is shown in Fig. 4.

Real data from the water supply network of Alexandria, a city in the north of Egypt, are used to illustrate and evaluate the models. This dataset was extracted from the Geographic Information System (GIS) office of Alexandria Water Company and was included in the Excel workbooks. It consists of 63423 data points, which cover the city of Alexandria with a total length of 3545.206 kilometres, taking into consideration different lengths of water pipeline (100–2000 mm), different types of pipeline materials (thermoplastic, concrete pressure pipes, and ferrous), Diameter, Hazen-Williams C, Flow (M^3^/H), Velocity (M/S), Head Loss Gradient (M/Km), Installation Year, Age (Years), Number of Breaks as input factors and failure rate as output, feature statistics of study dataset is presented in Table 2.

**Table 2:** Feature statistics of study dataset.

Name	Mean	Mode	Median	Dispersion	Min	Max	Missing
Length (Scaled) (m)	55.90	2	31	2.56	1	11,617	0 (0%)
Diameter (mm)	159.46	100	100	0.84	25	1500	0 (0%)
Material	3.50	5	5	0.49	1	6	0 (0%)
Hazen-Williams C	107.407	95.0	101.8	0.164	80.0	150.0	0 (0%)
Installation year	1985.57	1982	1983	0.01	1920	2019	0 (0%)
age	36.43	40	39	0.53	3	102	0 (0%)
Number of breaks	6.01	5	6	0.53	1	11	0 (0%)
Failure rate	0.238007	0.25	0.175	1.08604	0.00980392	3.66667	0 (0%)

The researchers preprocessed the data by replacing categorical variables like pipe material are encoded into numerical formats and replacing all missing values of attributes with the mean of the values because the most values in this case from a kind of numerical class attribute, the benefit of this pre-processing is to enhance the results of predictions for the predictive model and facility extract desired information from the dataset, as shown in Table 3.

**Table 3:** Data description.

	Variable	Type	Description
Input factors	Length	Numerical	The length of the pipe in meters(m)
	Diameter	Numerical	The diameter of pipe in millimeters
	Material	Numerical	The material of the pipe section, categorized as Numerical type
	Hazen-Williams C	Numerical	The relationship which relates the flow of water in a pipe with the physical properties of the pipe and the pressure
	Flow (m^3^/h)	Numerical	The average of flow of the pipe
	Velocity (m/s)	Numerical	The average of velocity of the pipe
	Head loss Gradient (m/km)	Numerical	Result of head loss calculated using Hazen-William's formula divided by total length of the pipe
	Installation Year	Numerical	The Installation Year of pipe
	Age (years)	Numerical	The age of pipes in years
	Number of breaks	Numerical	The number of total damages recorded on the pipe
Target	Failure rate	Numerical	The rate of water pipe failure

### Model development

Ensemble Learning Regression (ELR) is an ML approach that combines several models to improve prediction performance for nonlinear regression problems^36^. In this study, the researchers investigated three ensemble learning models: (A) Bootstrap Bagging (Bag) with Regression Trees (RT) Learners; (B) Least Square Boosting (LS Boost) with RT Learners; and (C) an optimizable ensemble method using Bayesian optimization. The model aims to improve the prediction performance by finding optimal values of "the minimum leaf size", "learning rate", "number of learners", and "number of predictors to sample" for the ensemble models’ optimizable hyperparameters.

**Figure 5:**
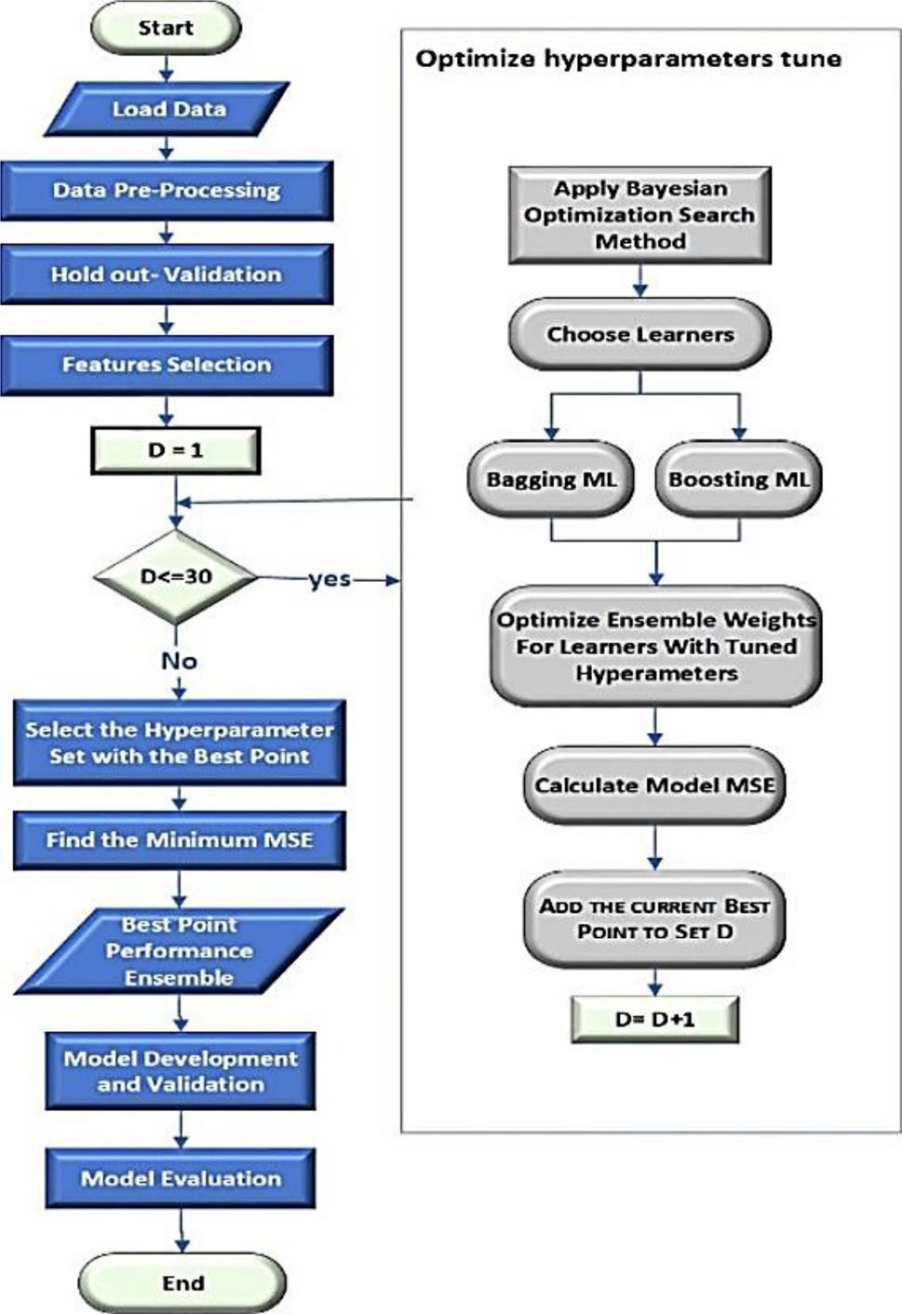
Proposed framework.

Models were developed to forecast the water pipe leakage on the basis of failure rate as the target based on more factors, such as "material", "diameter", and "length", etc. by using MATLAB version R2020a software^38^. The entire ensemble learning model process, which is represented using the flowchart in Fig. 5 and its stepwise implementation using MATLAB, is outlined as follows in the algorithm structure:

1. Load the data into the MATLAB software environment.
2. Preprocess the dataset.
  - Explore the dataset to get correlated features and types of variables.
  - Represented the correlated features.
  - Exitance: missing values and outliers.
  - Preprocess the missing values of data and categorical types of variables.
3. Modeling the dataset
  - Transform the dataset into an ensemble learning model format.
  - Identify the data set variable and the response.
  - Identify the percent of held-out using the holdout-validation process.
4. Apply the default Bagged and Boosting Ensemble tool in MATLAB for the data set.
5. Evaluate bagged and boosted ensemble methods fitting through the dataset.
6. Apply the Bayesian optimization process to identify the most relevant ensemble learning hyperparameters based on MSE values.
7. Build the final model by optimizing the LS-Boosted tree and bagged tree algorithm with Bayesian optimization.
8. Apply the resultant model to the entire throughout quality dataset.
9. Evaluate and report the predictive performance of the model.

### Experimental procedures

The researchers used three ensemble techniques, as presented in section "Proposed methodology for model development". The experiment results were implemented on an Intel (R) Core (TM) i7-10510U CPU @ 1.80 GHz and 2.30 GHz and the Windows 10 operating system. MATLAB software environment^38^ version R2020a software has been used for regression as a machine learning toolbox.

Configure using holdout-validation: 25%, because the dataset is large enough to avoid sample bias problems that will use previous research to focus the search space on the most promising values. Next, experiment using the boosting ensemble learning and bagged ensemble learning models. Configure the optimizable ensemble learning to use the maximum number of estimators at which the algorithm is ended ("number of learners": 8, and "a learning rate": 0.1). Following that, the researchers will examine what the algorithms have done, intending to determine which method is more likely to be efficient and how this efficiency varies by hyperparameter tuning, utilizing ensemble learning on our problem., finally, repeat the experiment in the optimizable ensemble to determine the optimal convergence with 30 iterations scoring: 'Mean Squared Error', as shown in Table 4.

**Table 4:** Performance of different decision tree-based models based on validation error.

	Bagged trees	Boosted trees	Optimizable ensemble
Minimum leaf size	8	8	29
Number of Leaners	30	30	272
Learning rate	–	0.1	0.85188
Optimized options	disabled	Disabled	Auto
validation	holdout-validation: 25%		

### Evaluation measurements

The efficacy of evaluation depends on which measure metrics are used; thus, it is essential to select metrics. Several metrics are often used to evaluate the performance of forecasting models.: root-mean-square-error (RMSE) given in (1), coefficient of determination (R2) given in (2), and mean square error (MSE) given in (3), mean absolute error (MAE) given in (4) are four evaluation metrics used in this paper to examine and evaluate the performance of the used machine learning methods^39–42^, shown in Table 5.

**Table5:** Statistical performance metrics description.

Statistic	Description	Equations	
RMSE	Always positive and its units match the units of your response	${\text{RMSE}}=\sum_{i=1}^{n} \sqrt{{\left({\widetilde{y}}_{i}-{y}_{i}\right)}^{2}}$	(1)
R2	Always smaller than 1 and usually larger than 0. If your model is worse than this constant model then R-Squared is negative	${R}^{2}=1-\frac{\sum_{i=1}^{n} {\left({y}_{i}-{\widehat{y}}_{i}\right)}^{2}}{\sum_{i=1}^{n} {\left({y}_{i}-{\overline{y} }_{i}\right)}^{2}}$	(2)
MSE	The MSE is the square of the RMSE	$\text{MSE }=\frac{1}{n}\sum_{i=1}^{n} {\left({{\widetilde{y}}_{i}-y}_{i}\right)}^{2}$	(3)
MAE	Always positive and similar to the RMSE, but less sensitive to outliers	$\text{MAE }=\frac{1}{n}\sum_{i=1}^{n} \left|{\widetilde{y}}_{i}-{y}_{i}\right|$	(4)

The mathematical expressions for these metrics can be denoted^42^ as follows where $n$ is the number of data simple, ${y}_{i}$ is the $i$ th measurement, and ${\widehat{y}}_{i}$ is corresponding prediction.

The model with the fewest average deviations for the same data are often chosen to use the fundamental assessment technique known as mean absolute error (MAE) is less sensitive to outliers. However, because they both amplify values with significant variances, the MSE (emphasize larger errors) and RMSE (easier interpretation of errors) are susceptible to outliers. They are therefore appropriate for assessing stability.

## Results and discussion

### Water- pipe leakage forecasting

In other literature, the kilometer where the leak appears can be used to computed the failure rate. In this case study, that information is not found so, this option is not chosen. The same pipe may have had more than one failure. However, the age has been considered as the difference between the Installation Year of the pipe and the constant year. Finally, we have worked with a dataset of dimensions 63423 × 10, where the information considered is exposed in the Table 3. In Table 6, the used inputs variables and methodologies are compared with other common inputs and methodologies considered in the reviewed literature.

**Table 6:** Comparison between Input Parameters and used methodologies in our case study and in reviewed literature.

Reference	Our case study	Variables (inputs)	Methodology
		Length, Diameter, Material, Hazen-Williams C, Flow (M^3^/H), Velocity (M/S), Head Loss Gradient (M/Km), Installation Year, Age (Years), Number of Breaks	Bagging Ensemble Technique, Boosting Ensemble Technique and Optimizable Ensemble Technique
^41^	(Jafari et al. 2021)	Diameter, Length, Installation Depth, Age, And Number of Pipe Failures	Linear Regression, Generalized Linear Regression, Support Vector Machine, Feed Forward Neural Network (FFNN), Radial-Based Function Neural Network (RBFNN), and Adaptive Neuro-Fuzzy Inference System (ANFIS)
^43^	(Giraldo-González and Rodríguez 2020)	Age, Length, Moisture content, Soil contraction and expansion potential, Precipitation, Land use, Valves, Hydrants, Previous failures	Linear, Poisson, Evolutionary Polynomial Regressions, Gradient-Boosted Tree (GBT), Bayes, Support Vector Machines and Artificial Neuronal Networks (ANNs)
^44^	(Sattar et al. 2019)	Pipe Length, Diameter, Material, and Previously Recorded Failures	Extreme Learning Machine (ELM), Artificial Neural Networks (ANN), Support Vector Machines (SVMs), and Non-Linear Regression (NNR)
^45^	(Motiee and Ghasemnejad 2019)	Material, Age, Length, Diameter and Hydraulic Pressure	Regression Models
^12^	(Kutyłowska 2019)	Length, Number of Failures, Failure Rate and Material	Support Vector Machines (SVM)
^46^	(Kutyłowska 2016)	Length, Diameter, Year of Construction of The Distribution Pipes and The House Connections	Support Vector Machines (SVMs) and Artificial Neural Networks (ANNs)
^47^	Shirzad, Tabesh, and Farmani 2014)	Age, Length, Diameter, Depth and Average Hydraulic Pressure	Artificial Neural Network (ANN) and Support Vector Regression (SVR)

With regards to the Table 1, Hazen-Williams C is the relationship which relates the flow of water in a pipe with the physical properties of the pipe and the pressure. It is very common in WDS to divide the system in segments based on the kind of pipelines(material). The cities have not at the same altitude, this factor can also be called as Height or Depth in other papers. In this paper, that information is not found so, this option is not chosen. Another factor that must be explained is Number of Breaks. In this case, calculated all the breaks in a pipe together. It is important to explain that Number of Breaks, once the pipe failure is repaired, the pipe has a different resistance than before So, this study tries to give a basic pattern to define a predictive model over WDS depending on the initial considerations over the problem.

### Ensemble learning models results

In this section more information about the tested models is exposed. The ELR was used for water pipe leakage forecasting via pipeline failure rate to assist in the decision-making process for the prioritization of water distribution networks rehabilitation measures. The researchers configured using the holdout-validation technique for large datasets to avoid sample bias problems by using 25% present held out-validation. The final model is trained using the full data set. The researchers conducted three sets of experiments as bagging ensemble technique, the boosting ensemble technique, and optimizable ensemble techniques as the Bayesian optimization approach was employed to fine-tune the hyperparameters of these ELR models, according to Table 7.

**Table 7:** Configuration of constructed optimizable ensemble models.

Optimizable hyperparameters	Range
Ensemble methods	[Bag, LS Boost]
Optimizer	Bayesian Optimizer
Acquisition function	Expected improvement per second plus
Minimum leaf size	[1–31711]
Number of learners	[10–500]
Learning rate	[0.001,1]
Number of predictors to simples	[1–10]
Iterations	30

The number of input predictors and samples is the range of optimizable hyperparameters for the ensemble model. The ideal hyperparameters for our study were chosen to use the Bayesian optimization technique from the ranges displayed in Table 7.

In this investigation, the loss function was the mean square error (MSE) between the objective values that were predicted and the actual values. The acquisition function used by the Bayesian optimizer is the expected improvement per second plus^37^ to ascertain the hyperparameter set for the following iteration. Water pipe leakage was predicted using the appropriate model, which had its set of hyperparameters optimized to minimize the upper-per-confidence interval of the MSE objective function.

**Figure 6:**
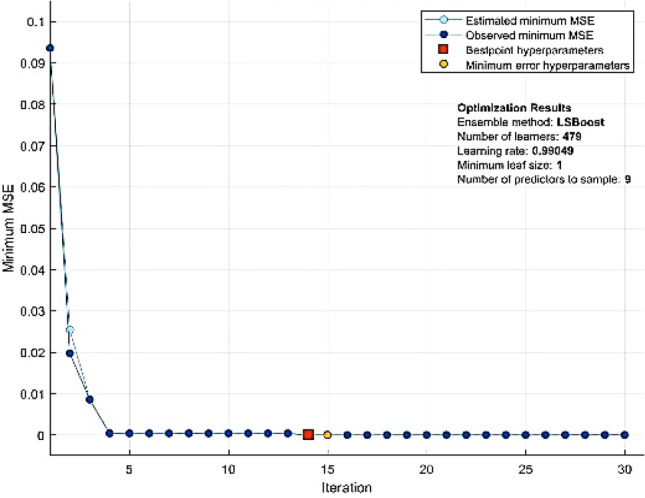
Performance curve of optimizable ensemble model.

The tuning process patterns and optimum hyperparameter values found using Bayesian optimization search are shown in Fig. 6, the curves in the figure represent the minimal hold-validated mean square error that results from identifying the ideal hyperparameter values, and shows that the best prediction of water pipe failure rate can be achieved by selecting the MSE function in the optimizable ensemble model, as shown in Table 7 This table shows the "Learning Rate", "Minimum leaf Size", and "Number of predictors to simples". In order to develop the proposed method, the optimizable ensemble-based model was over the Bayesian optimization method, as it has the lowest MSE.

**Figure 7:**
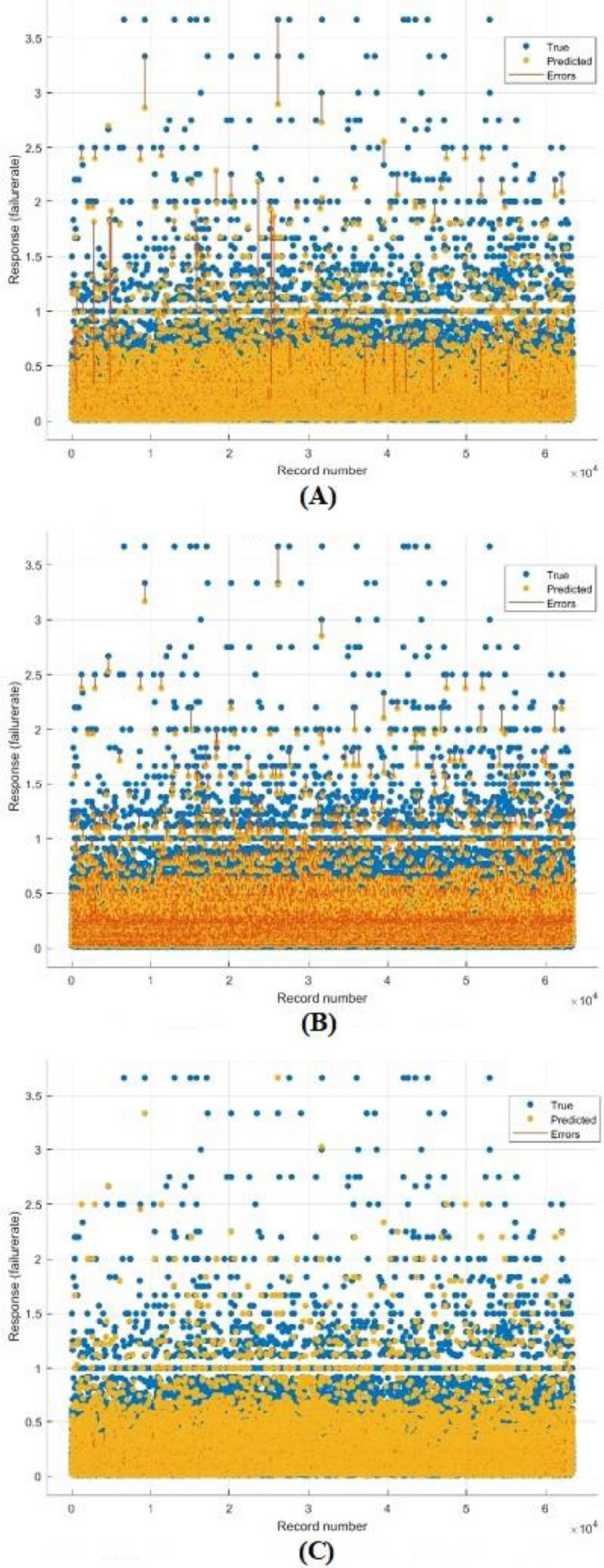
Response plots of failure rate: (**a**) bagged tree; (**b**) LSboosted tree; and (**c**) optimizable ensemble.

**Figure 8:**
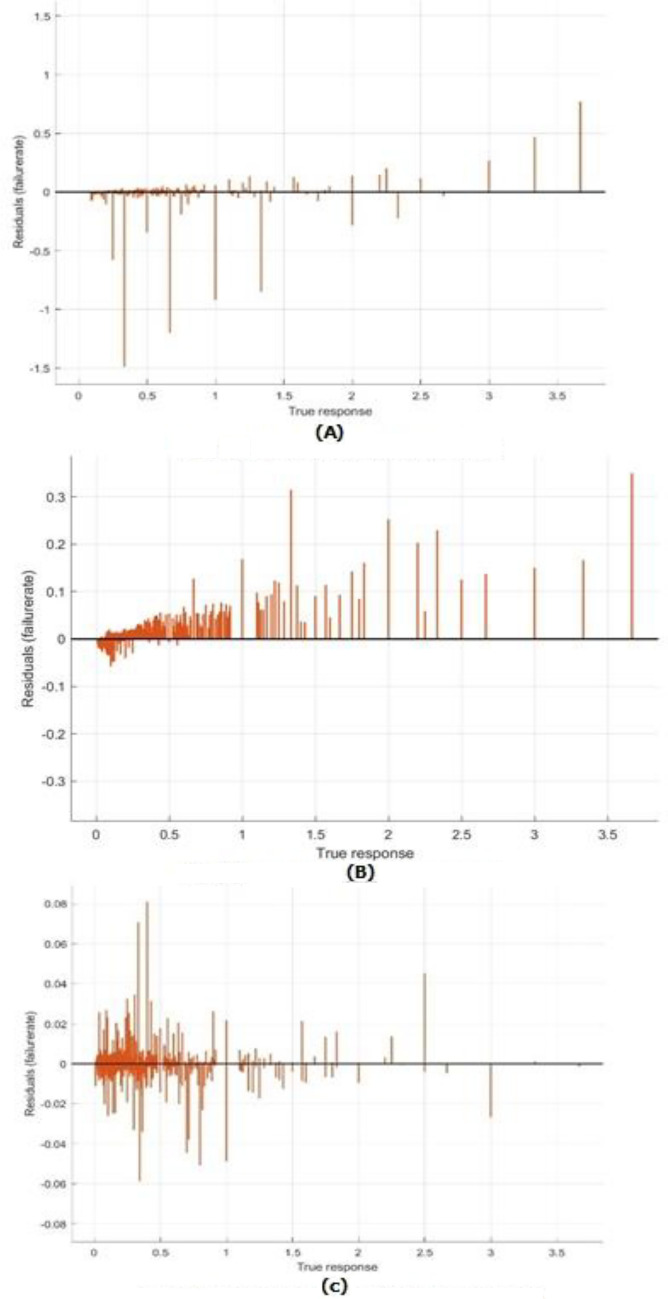
The Residuals plot of failure rate: (**a**) bagged tree; (**b**) LSboosted tree; and (**c**) optimizable ensemble.

**Figure 9:**
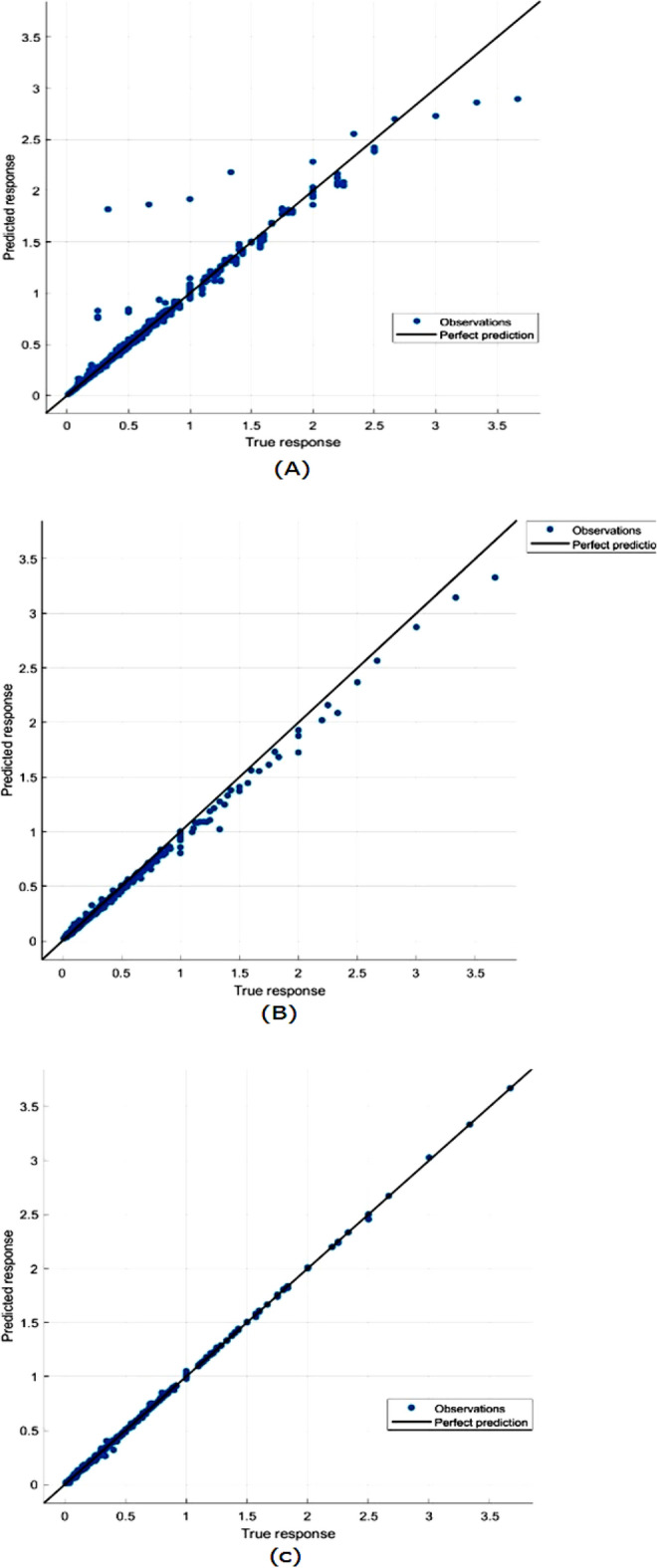
Predicted vs actuall plot of failure rate: (**a**) bagged tree; (**b**) LSboosted tree; and (**c**) optimizable ensemble.

Figure 7 showed response plots for the three models: the bagged tree ensemble technique, the boosted tree ensemble technique, and the optimizable ensemble technique, respectively. Figure 8 presents the Residuals plot of each model. Figure 9 demonstrates the predicted values comparing with actual plot of failure rate: (a) bagged tree; (b) LS boosted tree; and (c) optimizable ensemble.

In Fig. 9, shown the predicted values versus actual response have been plotted, showing that most of the values match, except for a few data points where the true and expected values diverge significantly. The breadth of the band for residual values in the residuals plot, as shown in Fig. 8, is constant with a few exceptions. The model gains are stable across all regression models due to the performance of test data in the same. In Fig. 7, versus actual values of water pipe leakage forecasting via pipeline failure rate and demonstrates that all the developed models scored high R2. The results also show that there is no high variation between predicted and actual values, and there are no outliers.

The study used a set of mathematical validation equations to evaluate each model's performance. The evaluation matrices demonstrated that bagged trees has RMSE 0.03195, MAE 0.0041853, and R2 0.98. However, LS boosted trees has RMSE 0.022654, MAE 0.014829, and R2 0.99. Optimizable Ensemble, on the other hand, has RMSE 0.00231, MAE 0.00071513, and R2 as 1, presented in Table 8. The results showed that all models could forecast the failure rate of water pipes.

**Table 8:** Comparison of the Three Intelligent Models.

Results	Bagged trees	Boosted trees	Optimizable ensemble
RMSE	0.03195	0.022654	0.00231
R2	0.98	0.99	1
MSE	0.0010208	0.00051322	5.34E-06
MAE	0.0041853	0.014829	0.00071513

Table 8 compares the RMSE, *R*2, MSE, and MAE of the minimum correlation bagged ensemble learning model, LS boosted ensemble learning model, and optimizable ensemble learning model by hyperparameters. Experiments show that the maximal correlation optimizable ensemble learning model can achieve the best prediction effect, and RMSE, *R*2, MSE, and MAE are 0.00231, 1, 5.34E−06, and 0.00071513 respectively. Compared with the bagged tree and LS boosed tree ensemble learning method and optimizable ensemble model combination, the proposed model also achieved better results. It is observed that the developed ELR models have satisfied.

### The computational complexity

The computational complexity of the ensemble approach is an additional essential aspect to consider. The main disadvantage of the optimizable ensemble due hyperparameters tune is their complexity. They are much time-consuming to training (training time) than boosted and bagged tree. They also require more computational resources. Also, provided the time complexity of the methods for prediction speed (Observation/Second). Prediction speed measure via (obs/s) refers to number of observations processed per second. It's inverse would be the time taken for one prediction in seconds.

**Figure 10:**
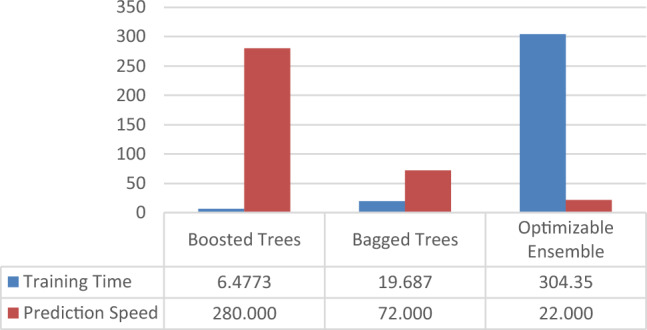
Comparison of Training time (sec) and prediction speed (obs/sec) plot for the three algorithms.

The complexity of each algorithm is shown in Fig. 10, where the vertical axis represents the complexity on algorithmic scale via prediction speed and training time. The result showed that Boosted Tree provides the best result for prediction speed (280000 Obs/s) in relation to bagged tree (72000 Obs/s), and optimizable ensemble (22000 obs/s). the researchers observed that the optimizable ensemble model had the highest predictive capacity. However, due to its high complexity, the prediction speed of the optimizable ensemble model is highly dependent on the hardware used.

According for, Table 9 and Fig. 10, that the proposed algorithm has the best prediction rate of all methods with an opposite order of complexity. The complexity of the hyperparameters tune optimizable ensemble, which achieved the highest accuracy, is number one in orders of training time complexity. Of course, the complexity of the ensemble learning methods increases with hyperparameter tune optimization; hence, the training time of the boosted and bagged tree methods is less than that of the proposed method. Thus, the prediction process using optimizable ensemble is time-consuming than other algorithms. This can be an issue for large datasets.

**Table 9:** Complexity comparison of the three algorithms.

	Boosted trees	bagged trees	Optimizable ensemble
Prediction speed	280,000 obs/s	72,000 obs/s	22,000 obs/s
Training time	6.4773 s	19.687 s	304.35 s

### Comparison of different machine learning models

According for Table 6 found that SVM, ANN, LR more used methods to compare and apply for our problem in the reviewed literature. Further evaluation for the developed ELR models has been performed with results presented in Table 4. While the ELR model is considered a good, it compared to some of ML methods to improve the applicability of the model and confirm they have good prediction ability.

### Model setup for machine learning models

Concerning the SVMs, the capacity (C), gamma (γ), and epsilon (ε) is the parameters that must be defined, shown in Table 10 as SVM-L model, and Table 11 as SVM-RBF model.

**Table 10:** SVM-L model parameters.

SVM-L model 43	
Gamma	–
Capacity(C)	2
Epsilon (ε)	0.1
Number of support vectors (localized)	2
Cross-validation error	0.008

**Table 11:** SVM-RBF model parameters.

SVM-RBF model 48	
Gamma	0.333
Capacity(C)	3
Epsilon (ε)	0.2
Number of support vectors (localized)	56 (46)
Cross-validation error	0.081

Regarding the ANNs, the number of input layers, the number of hidden layers, the neurons in the hidden layers, the training cycles, the learning rate, and the activation function are the parameters that must be defined, shown in Table 12 as ANNs model parameters.

**Table 12:** ANNs model parameters.

ANNs model parameters 45	
Input layers	10
Hidden layers	2
Hidden layer neurons	8
Training cycles	2000
Learning rate	0.2
Activation function of hidden layers	Sigmoid
Activation function of the output layer	Sigmoid

### Comparison of different machine learning models results

Consider now the performance of the developed ELR models in comparison with other machine learning methods, namely support vector machines (SVMs), Artificial Neural Networks (ANN), and Linear Regression (LR), as presented in Table 13.The developed ELR models show better performance than other machine learning models applied on the same dataset in terms of RMSE, MSE, MAE, and R2 values, All tests different machine learning models were completed in Orange Data Mining with an Intel(R) Core (TM) i7-10510U CPU @ 1.80GHz 2.30 GHz,16 GBRAM, PC.

**Table 13:** Comparison of different machine learning models.

Results	Optimizable Ensemble	SVM-L	SVM-RBF	ANN	LR
RMSE	0.00231	0.251	0.073	0.056	0.163
R2	1	0.059	0.920	0.953	0.601
MSE	5.34E-06	0.063	0.005	0.003	0.027
MAE	0.00071513	0.166	0.060	0.023	0.089

## Conclusion

Using artificial intelligence-based techniques for solving decision support and engineering issues are common in today's world. This work presents a thorough and insightful investigation of the use of ensemble models on real dataset in water pipe leaking. Several common ensemble models and hyperparameter tuning strategies are being investigated to help researchers and practitioners use ensemble learning methods for data-driven predictions. Specifically, three ensemble models were studied.; optimization ensemble method, boosted tree ensemble learning and bagged tree ensemble learning, while evaluating the model performance using the RMSE, MSE, MAE, and R2 values for the failure rate as evaluating parameters.

This paper presented a hyperparameter tuning optimization for models of Bayesian optimization-based ensemble learning real-world dataset is used in experiments to evaluate the effectiveness of various ensemble models and optimizable ensemble methods, as well as to offer useful examples of hyperparameter optimization. In light of the approach outlined in "dataset generation" and "ensemble learning algorithms development", the generated dataset is entered into various ensemble learning models, including the bagging ensemble technique, and the boosting ensemble technique as homogeneous ensemble, and the optimizable ensemble technique. Hyperparameter tuning methods are employed to enhance the learning procedures to predict water pipe leakage based on the failure rate. 

This study was conducted to develop an optimization-based ensemble learning model with Bayesian optimization for water pipe leakage forecasting via pipeline failure rate. The developed model applied to a real dataset of water pipe leakage from AWCO in Egypt and compared it to state-of-the-art ensemble learning methods. In light of the outcomes that were achieved, it was shown the three models had shown acceptable performances, the optimizable ensemble model was the most efficient, showing an RMSE of 0.00231 and an R2 of 1. These parameters were calculated by comparing actual and predicted cases during hold-validation. Our study demonstrates that the proposed model has excellent accuracy and high application value and shows unique advantages.

This paper will help decision-makers in the decision-making process, through developing an optimization-based ensemble learning method that can optimize weights and tuning hyperparameters of ensemble learning methods in water pipe leakage forecasting as pipeline failure rate. For future research, the researchers will integrate this model that developed into an internet of things (IoT) system.

## Data Availability

The data that supports the findings of this study is available from Alexandria Water Company. Restrictions apply to the availability of these data, which were used under license for the current study and are not publicly available. However, data are available from the corresponding author upon reasonable request and with permission from Alexandria Water Company.
